# The Use of Digital Technologies in the Promotion of Health Literacy and Empowerment of Informal Caregivers: Scoping Review

**DOI:** 10.2196/54913

**Published:** 2024-04-29

**Authors:** Suzete Soares, Louíse Viecili Hoffmeister, Maria de Fátima Fernandes, Adriana Henriques, Andreia Costa

**Affiliations:** 1 Nursing School of Lisbon Lisbon Portugal; 2 Family Health Unit Carnide Quer North Lisbon Health Center Cluster Lisbon Portugal; 3 Nursing Research, Innovation and Development Centre of Lisbon (CIDNUR) Nursing School of Lisbon Lisbon Portugal; 4 National School of Public Health NOVA University Lisbon Portugal; 5 Community Care Unit Integrar na Saúde Administração Regional de Saúde de Lisboa e Vale do Tejo Lisbon Portugal; 6 Institute of Environmental Health Faculty of Medicine of the University of Lisbon Lisbon Portugal

**Keywords:** health literacy, empowerment, digital technology, informal caregiver, family caregiver

## Abstract

**Background:**

Informal caregivers (IC) play an important role in the community as health care providers for people who are dependent on self-care. Health literacy contributes to empowerment, better care, and self-management of one’s own health and can be developed using digital technologies.

**Objective:**

This study aims to map scientific evidence about the use of digital technologies to promote health literacy and the empowerment of ICs.

**Methods:**

We conducted a scoping review following the Joanna Briggs Institute methodology. The CINAHL, MEDLINE, Scopus, and PubMed databases were searched to find primary studies on the theme. Inclusion criteria were based on the Population, Concept, and Context logic. To be selected for analysis, studies must have involved informal or family caregivers aged ≥18 years who provide care to dependent persons and who have access to the internet and digital devices (computer, smartphone, and tablet). A total of 2 independent researchers (SS and LVH) performed the screening process. This study is part of a main project that was approved by the Ethics Committee for Health of the Regional Health Administration of Lisbon and Tagus Valley (reference 058/CES/INV/2022).

**Results:**

A total of 9 studies were included in the review. The analysis of the studies showed that ICs use digital tools, such as computers and smartphones, with smartphones being the preferred tool. ICs use the internet to access information; manage home tasks; communicate with relatives, their peers, and health care professionals; and take part in forums. Due to difficulties in leaving their houses, forums are highly valued to preserve human connections.

**Conclusions:**

The use of digital technologies to convey clear, objective, reliable, and accessible information is a strategic action for promoting health literacy and for contemplating the variable care needs of ICs. By working with ICs in the development of new technologies, researchers are building a new tool that meets ICs’ needs.

## Introduction

### Background

Population aging is a social challenge worldwide. As life expectancy increases, the incidence of chronic and incapacitating diseases also increases [1]. The high levels of dependence and the complex management of health status raise awareness of the increasingly relevant role of informal caregivers (ICs) in terms of care and health promotion of their relatives [2]. The ICs, defined as *someone who provides nonremunerated care to a person with a long-term illness, disability or other health need, or long-term care, outside a professional or formal framework*, are considered essential both to the care of people in the community and to the economy of European Union countries [3]. ICs are more and more important to patients as well as to health care professionals [4]. They play a central role in the planning, training, and provision of services to people with care needs [2,3]. In practical terms, ICs collaborate in providing health care at home to people who show an impairment in self-care, activities of daily living, and instrumental activities of daily living. The needs change over time, as does the level of dependency of the person cared for [5].

In most situations, ICs are not prepared to play this role. This transition in caregivers’ lives brings out feelings of insecurity due to the unknown and the lack of knowledge and skills to ensure that the person cared for is given proper care [5]. This way, ICs’ existing and acquired knowledge is extremely important and becomes necessary for the implementation of interventions destined to promote the development of skills and the involvement of relatives in patient trajectories to improve patient outcomes [2-4].

A vital point in health policies is the investment in the health literacy (HL) of ICs. HL is central to empowering people, their families, and communities, promoting greater control over decisions and actions affecting their health [6]. HL is defined as the ability to access, understand, evaluate, and apply information about health care, disease prevention, and health promotion to maintain and promote quality of life during the life course [7].

Through HL development, conditions are created for individuals to gain knowledge and skills, make informed decisions, and feel motivated to adopt a behavior that improves their health status and well-being [8].

Considering that HL is a health determinant, mediator, and moderator, it is important to ensure that citizens access reliable, useful, and updated health information to help them make the best decisions about their personal health, their family’s health, and the community’s health [9-11]. Proper access to information allows to promote and increase citizen empowerment so that they participate in their health care, leading to shared responsibility and informed decision-making [12].

As an agent, the health care professional plays a central role in effective communication and in conveying reliable information to the population. User-relative–health care professional communication significantly affects health outcomes and user satisfaction concerning health services [13]. Digital technologies have created an opportunity for health professionals and health organizations to directly communicate with many people in real time. This digital revolution in communication allows to customize information, help people set health targets, and interact in real time [1].

Information and Communication Technologies (ICTs) is the set of technologies and equipment that, in an integrated manner, allow working and communicating information, including computers and the respective applications, the internet, and telecommunications [14]. They are part of the citizens’ routine, with an increasing use of educational platforms. The internet is considered a privileged means of interaction with the population that needs health care [1].

ICTs improve the quality of life of older adults and their caregivers and their access to quality care, contributing to improving the social lives of caregivers and decreasing their isolation via social activities and intergenerational relationships [15]. These aspects contribute to balanced physical, mental, and emotional health and to a decrease in depressive symptoms and sadness. Digital technologies are considered *a key component and facilitator of sustainable health systems and universal health coverage* [16]. Digital technology is a strategy that can promote accessibility to health care for all citizens. Digital means can be used to increase access to reliable, useful information and to strategies that meet the needs of the highest possible number of ICs, whether in real time or not [1]. However, accessing and handling these technologies requires digital literacy, which is one of the barriers identified by studies in certain groups considered vulnerable, such as older adults. Digital HL is the ability of citizens to use digital platforms to manage their health, validate web-accessible health information, and communicate with health professionals [17].

### Objectives

In Portuguese literature, there are only a few scientific studies conducted by nurses that refer to the use of digital technologies as a resource to empower dependent people and family caregivers [1]. The need to know if dependent people and their ICs have access to digital technologies and use them when they have health needs gave rise to the following research question: “Which digital technologies are used for promoting Health Literacy and empowering the Informal Caregiver?” For the mapping, we used the following guiding questions: “Do the Informal Caregivers have access to digital technology?” and “Do the Informal Caregivers use digital technologies to improve their health literacy and empowerment concerning the care of the person cared for?” To answer these questions, this review aims to map the scientific evidence regarding the use of digital technologies to promote HL and empower ICs.

## Methods

### Overview

This is a scoping review conducted according to the methodology recommended by the Joanna Briggs Institute (JBI) [18]. Scoping reviews are used to identify knowledge gaps, enhance knowledge described in the literature, clarify concepts, or investigate research conduct [19].

The theme was searched in the JBI Database of Systematic Reviews, CINAHL, MEDLINE, Scopus, and PubMed, and no systematic review was found for this same theme. The inclusion criteria were based on the Population, Concept, and Context logic: the Population included all informal or family caregivers aged ≥18 years who provide care to dependent persons and who have access to the internet and digital devices (computer, smartphone, and tablet).

The search was conducted from April 4 to 18, 2022, and included primary qualitative and quantitative studies and mixed method studies in English, Portuguese, French, and Spanish, during 5-year period between January 2017 and December 2021, to obtain the most recent studies published on this theme. Key terms and inclusion criteria were used as a strategy to identify papers that were relevant to the search.

### Study Selection, Data Extraction, and Analysis

According to the JBI’s recommendations, the search strategy was performed in 2 steps [18]. There was an initial search of the electronic platform EBSCO, in particular, MEDLINE and CINAHL, with the natural keywords informal caregiver; family caregiver; health literacy; empower; digital technology; and community, following the search for the indexing term MH “Empowerment.” Subsequently, we carried out an analysis of the words used in the title, the abstract, and the terms indexed as well as the keywords presented in the description of each searched article. We then carried out a second survey in which the indexing terms and keywords were searched in MEDLINE (PubMed), CINAHL (via EBSCO), MEDLINE (via EBSCO), and Scopus (Textbox 1).

A total of 2 independent reviewers (SS and LVH) analyzed the relevance of papers using the information included in the title and abstract, considering that the study population must be defined and the goal must be associated with digital tools.

It was necessary to retrieve the papers after reading the abstract. Full papers were obtained for all studies with the inclusion criteria. A table was filled with the defined criteria, considering the goals and the results of the study that would answer the research question. After reading the full text, 2 papers showed a divergent opinion. This situation was discussed and resolved without the need to speak to a third reviewer.

**Figure 1:**
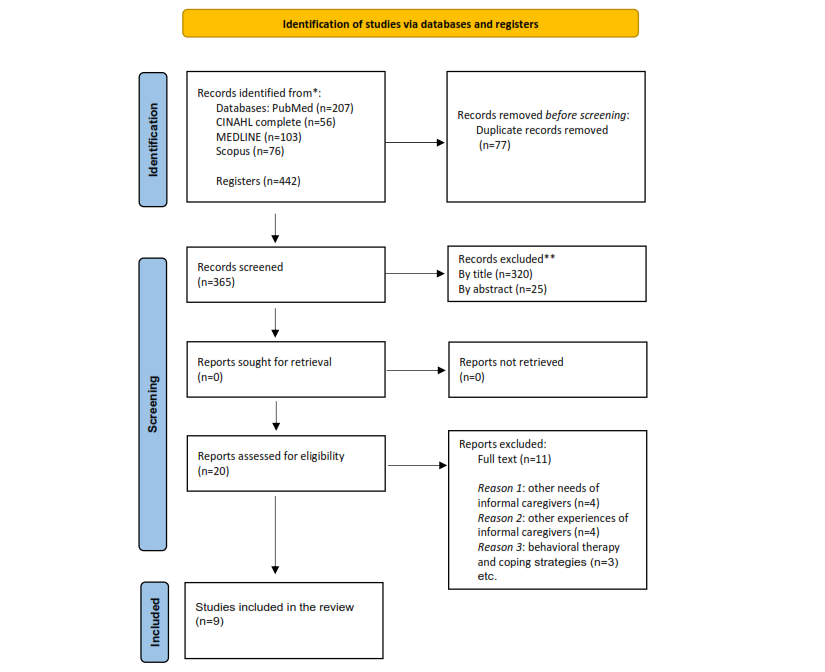
PRISMA (Preferred Reporting Items for Systematic Reviews and Meta-Analysis) flowchart.

The screening process identified 442 studies. Of the 442 studies, 77 (17.4%) were duplicated and so were excluded. Of the remaining 365 studies, 320 (87.7%) were excluded for their titles and 25 (6.8%) for their abstracts, based on the inclusion criteria that had determined their eligibility. In the second step, there were 20 papers for full-text review. Of the 20 papers, 11 (55%) were eliminated because of the following reasons: 4 (20%) because their goals did not relate to the technological needs of ICs but to the person cared for; 4 (20%) because they were about another type of nontechnological experience; and 3 (15%) because they were about behavioral therapies and coping strategies. Figure 1 [20] shows the PRISMA (Preferred Reporting Items for Systematic Reviews and Meta-Analyses) flowchart for the identification and selection of the studies.

A data collection instrument was made to extract information from the selected studies, including the following items: author, country, year of publication, study goal, study type and methodology used, population, sample, types of interventions, main results, and conclusions. The results were analyzed based on their content and organized according to the research question and goals.

**Search strategy according to database searched.**

**MEDLINE (via PubMed)**

- (((informal caregivers) OR (family caregivers) AND (community) AND ((“health literacy”) OR (empowerment) OR ((digital education) OR (digital technology) OR (digital era) OR (digital platforms) OR (digital sources) OR (Information and communication technology))) in the last 5 years

**CINAHL complete (via EBSCO)**

- S1 informal caregivers
- S2 family caregivers
- S3 S1 OR S2
- S4 community
- S5 health literacy
- S6 empowerment
- S7 MH“empowerment”
- S8 empower*
- S9 digital technology
- S10 digital era
- S11 digital health literacy
- S12 digital sources
- S13 digital education
- S14 digital platforms
- S15 Information Communication Technology
- S16 S5 OR S6 OR S7 OR S8 OR S9 OR S10 OR S11 OR S12 OR S13 OR S14 OR S15
- S17 S3 AND S4 AND S16

**MEDLINE (via EBSCO)**

- S1 informal caregivers
- S2 family caregivers
- S3 S1 OR S2
- S4 community
- S5 health literacy
- S6 empowerment
- S7 MH“empowerment”
- S8 empower*
- S9 digital technology
- S10 digital era
- S11 digital health literacy
- S12 digital sources
- S13 digital education
- S14 digital platforms
- S15 Information Communication Technology
- S16 S5 OR S6 OR S7 OR S8 OR S9 OR S10 OR S11 OR S12 OR S13 OR S14 OR S15
- S17 S3 and S4 and S16

**Scopus**

- (TITLE-ABS-KEY [“informal caregivers”] AND PUBYEAR>2016) OR (TITLE-ABS-KEY [“family caregivers”] AND PUBYEAR>2016 AND PUBYEAR<2023) (TITLE-ABS-KEY [community] AND >2016 AND PUBYEAR<2023) (TITLE-ABS-KEY [“health literacy”] AND PUBYEAR>2016 AND PUBYEAR<2023) OR (TITLE-ABS-KEY (“empower*”) AND PUBYEAR>2016 AND PUBYEAR<2023) OR (TITLE-ABS-KEY [“empowerment”] AND PUBYEAR>2016 AND PUBYEAR<2023) OR (TITLE-ABS-KEY [mh “empowerment”] AND PUBYEAR>2016 AND PUBYEAR<2023) OR (TITLE-ABS-KEY [“digital technology”] AND PUBYEAR>2016 AND PUBYEAR<2023) OR (TITLE-ABS-KEY [“digital era”] AND PUBYEAR>2016 AND PUBYEAR<2023) OR (TITLE-ABS-KEY [“digital sources”] AND PUBYEAR>2016 AND PUBYEAR<2023) OR (TITLE-ABS-KEY [“digital health literacy”] AND PUBYEAR>2016 AND PUBYEAR<2023) OR (TITLE-ABS-KEY [“digital education”] AND PUBYEAR>2016 AND PUBYEAR<2023) OR (TITLE-ABS-KEY [“digital platforms”] AND PUBYEAR>2016 AND PUBYEAR<2023) OR (TITLE-ABS-KEY [“information and communication technology”] AND PUBYEAR>2016AND PUBYEAR<2023) (TITLE-ABS-KEY (*#3* AND *#4* AND *#16*) AND PUBYEAR> *2016* AND PUBYEAR< *2023* AND (LIMITTO [LANGUAGE, *“English”*] OR LIMIT TO [LANGUAGE, *“Spanish”*] OR LIMIT TO [LANGUAGE, *“French”*] OR LIMIT TO [LANGUAGE, *“Portuguese”*])

### Ethical Considerations

This study is part of a main project that was approved by the Ethics Committee for Health of the Regional Health Administration of Lisbon and Tagus Valley (reference 058/CES/INV/2022).

## Results

### Studies Characteristics

In total, 9 studies were included in this review after the search. According to the JBI’s guidelines, after the selection, the studies were organized based on their research question and goals (Tables 1 and 2).

**Table 1:** Summary of study characteristics.

Paper title	Authors, country, and year	Goal	Type of study and methodology	Population and sample
What is “care quality” and can it be improved by information and communication technology? A typology of family caregivers’ perspective	Leslie et al [21], Canada, 2020	To determine how ICTs^a^ can support family caregivers who play the caregiver role	Qualitative study Method Focus group	FC^b^ of older people Sample: 25
Effect of an innovative model of complexity care on family caregiver experience: qualitative study in family practice	Nickell et al [22], Canada, 2020	To learn about the experiences of FC of older people with complex needs, using the Interprofessional Model of Practice for Aging and Complex Treatments	Qualitative study Method Individual interviews	FC of older people with complex needs Sample: 20
Building a Research Roadmap for Caregivers Innovation: Finding from a Multi-Stakeholder Consultation and Evaluation	Egan et al [23], Scotland, 2021	To explore a future roadmap for innovation from IC^c^ participation	Mixed study Method Interview Questionnaire	ICs Sample: 112 Professionals and researchers Sample: 62
The care capacity goals of family carers and the role of technology in achieving them	Leslie et al [24], Canada, 2020	To identify the goals of FC when caring for older adults and how technology can help achieve those goals	Mixed study Method Focus group Questionnaire	FCs Sample: 25
Mobile Support for Older Adults and Their Caregivers: Dyad Usability Study	Quinn et al [25], United States, 2019	To determine the usability of a mobile app within the older population and in their relationship with ICs	Observational study Method Questionnaires	Older people and ICs Sample: 24 (dyad 12)
Preferences for using a Mobile App in Sickle cell Disease Self- management: descriptive Qualitative study	Mayo-Gamble et al [26], Canada, 2020	To explore health preferences for using an app in the process of facilitating the self-management of adults with sickle cell disease and their caregivers who live in urban and rural communities	Qualitative study Method Focus group	Adults with sickle cell disease and caregivers Sample: 43
A Digital Mobile Community App for Caregivers in Singapore: predevelopment and Usability Study	Lwin et al [27], Singapore, 2021	To provide a clear understanding of the implementation along with a usability study to gauge user opinion of the “Caregiver’s circle” app within Singapore	Qualitative study Method In-person interviews Questionnaire	ICs Sample: 103
Improving the Quality of Life of Family Caregivers of People with Alzheimer’s Disease through Virtual Communities of Practice: A Quasiexperimental Study	Romero-Mas et al [28], Spain, 2021	To describe the relation between the quality of life of ICs of people with Alzheimer disease and their participation in a VCoP^d^ (virtual community with the exchange of knowledge and an emotional support and collaboration culture) To determine the impact of ICs’ HL^e^ in the quality of life and involvement in the VCoP	Quasiexperimental study Method Phone calls and in-person contact Focus group Evaluation scales Control group with and without VCoP intervention Questionnaire	ICs of people with Alzheimer disease Sample: 38 before the test and 37 after the test
Patient Portals as a Tool for health Care Engagement: A Mixed-Method Study of older Adults with Varying Levels of Health Literacy and Prior Patient Portal Use	Irizarry et al [29], United States, 2017	To explore attitudes in relation to choosing the portal and its utility as a tool to involve health care with different levels of HL	Mixed-methods study Method Phone interview Focus group	Older people Sample: 100

^a^ICT: Information and Communication Technology.

^b^FC: family caregiver.

^c^IC: informal caregiver.

^d^VCoP: virtual community of practice.

^e^HL: health literacy.

**Table 2:** Summary of study results.

Types of interventions	Main findings	Conclusion
Intervention made with 10 focus groups from May 2017 to August 2018. Each session took 2 hours. Bottom-up approach with thematic content analysis.	Technologies that are only focused on the task can lose their value as they lose the capacity to provide information that is relevant to caregivers’ needs. ICTs^a^, as the intermediary for an improvement in quality of life and as providers of relevant information that are enabled with knowledge and caregiver needs’ change. Smartphones can be an extension of access to software.	ITC product development supported by ICs^b^ should focus on human relationships and expand a facilitating communication, allowing their participation in decision-making and allowing them to express their concerns and goals. Technology appears as a support to receive information that is relevant to caregivers’ needs and to establish human connections.
Individual interviews with 13 family caregivers about the caregiver role and their (physical and emotional) well-being. The patient and the caregiver are encouraged to play a more active role in the process of their disease by raising questions and discussing actions.	Caregivers reported that they no longer felt lonely in this role because they were given basic information about the disease as well as existing resources and equipment. They felt recognized and heard; they were able to express their uncertainties, stories, and suggestions, increasing their commitment to caregiving. They searched the internet.	Involving ICs as part of the multiprofessional team increases their perception and understanding of the caregiver role and their trust in their ability to perform this role and facilitates their empowerment.
A mixed approach was used: A 10-minute multisector consultation from June 15, 2020, to September 30, 2020. Web-based questionnaire on social media.	In total, 108 of the 112 (96%) ICs use digital technology. The hybrid approach (both in person and web based) can work for caregivers. The experience of ICs in collaborating with universities to work via multiple communication channels should be valued. Deep knowledge of needs and existing gaps allows one to contribute to technological innovation to overcome existing technological barriers and learn what the facilitating mechanisms are. The ICs mentioned the need for improved financial, emotional, psychological, training, and educational support.	A technological approach in the following areas is required for the health and well-being of ICs: information, monitoring technology, and communication with other ICs and professionals. The experience of ICs in collaborating with universities to identify priorities and actions that speed up searches and future political decisions about significant and innovative solutions should be valued.
Sequential method, focus group, and web-based questionnaire. In total, 10 focus groups with 25 family caregivers. The intervention took place from May 2017 to August 2018. First part: discussion of targets and technological solutions. In what they think technology can help them. Second part: web-based questionnaire about 7 fields: physical health, mental health, well-being, social connection, education, employment, and finances.	Technology maintains the ability to care and allows ICs to develop coping strategies, guide themselves, and socialize. Technology is an intermediary that connects ICs to information support and other caregivers. Key targets for ICs are to reinforce and preserve their ability to provide care.	Technology is well positioned to find the best self-care to facilitate the connections needed for a social life. Technological targets and suggestions should imply that the understanding of care as a source of overload was transformed into a more resilient, sustainable caregiving model. Technology can help promote such resilience but can be limited to the role of an intermediary that connects family caregivers to information supports and peers.
Participants completed a skill evaluation questionnaire and downloaded an app to their smartphones or computers that was used for a month. Then, participants completed 2 questionnaires that evaluated app features and esthetics and their relationship with the app. App features: user profile, family health history, health information, receiving studies based on their health profile, and establishing a relationship with their caregivers.	Study results showed normal levels of digital competence for the older adults and high levels for the ICs. Older adults use their smartphones to make calls (9/12, 75%) and read emails (7/12, 58%). They access the internet (4/12 33%) but on their computers. ICs use their smartphones for calls, SMS text messages, emails, and the internet equally (11/12, 92%). They access the internet via their phones. This study concluded that 50% (6/12) of ICs want to use the app to manage the appointments and clinical information of the person cared for and to access specific information that allows them to share and discuss to commit to the caregiving; they believe that the app’s esthetic dimension is important.	Technologically experienced caregivers play an essential role in showing the benefits of technology for supporting care provision for older adults. There were high levels of use of technology among the older adults and caregivers, but there was only an average use of the mobile app. Additional training is recommended for the older adults and caregivers, including behaviors directed toward keeping digital health records.
In total, 5 community listening sessions were made with 1 urban and 1 rural community. Each session took 2 hours. A questionnaire about demographics and access to technology was applied. Where they searched for information about self-care in relation to the SCD^c^ and what was their satisfaction level with the search for and support about management and resources. A total of 7 aspects were evaluated: self-management information, such as receiving information, which information they wish to receive, changes in disease management, support types, barriers to and facilitators for the use of apps, and mobile app preferences.	Participants are receptive to using the app to self-manage the disease. A mobile app reduces the information access barrier. In rural communities, the app increases ICs’ access to resources. The internet is the reported source to learn about self-management techniques and receive information, reinforcing the importance of reliable websites. ICs want emotional support, information support from the family, and follow-up from health care professionals. Positive feedback about the app included easy configuration and a good interface. Barriers: participants were not comfortable using the internet because they struggled to identify relevant, reliable information. The notification system, information trackers, and the fact that they can communicate with their health care professionals and caregivers were aspects valued by patients.	The results can be used to develop a patient-centered health app that is easy to use to facilitate disease self-management, thereby increasing access to resources by relatives that live in rural communities.
A predevelopment survey was made about the following issues: care, support provided, and what they would like in a caregiving mobile app. Identifying the needs of ICs and the gaps in web community networks. Demographics about the health of the person cared for and about the ICs’ physical and mental health. What is the level of use of digital means when searching for information and support. A total of 32 caregivers completed a web-based questionnaire and in-person interviews, followed by a usability test.	ICs said they liked using the app. They said it was useful, easy to use, and helpful to improve the quality of life because they included multiple resources: a public forum for discussions with the community and other ICs in the same region without ever leaving home and a market to purchase and sell material and equipment required for caregiving. Including many resources that caregivers need daily in an easy-to-use app allowed them to save time and helped browse without any issues. The use of smartphones created an opportunity for the caregiving community to use technology in a useful way. The app included caregivers’ ideas, which created an app that facilitated caregiving. As to concerns about safety and security, trust would increase if the app were supported by a renowned organization. ICs have suggested that the app should include a resource that would help with mental health, namely, relaxation techniques, motivational quotes, and guides that would remind them to take care of themselves.	Caregivers enjoyed the “Caregivers’ Circle” and were confident that this app could help them improve their quality of life. Including many resources that caregivers need daily in 1 app can help save time and help them live without problems.
The study took place between July 2017 and April 2018. Previous contact was made with the AFMADO^d^ association, and explanatory sessions were held (individual and group). In total, 2 groups were created, 1 with and 1 without health care professionals. Intervention: developing an app based on the CoP^e^ theory, with space for chatting and a member file with information about each member. The following aspects were evaluated before and after the VCoP^f^ intervention: quality of life, HL, and the Barthel scale associated with the Spanish population.	QoL^g^ was 66.6 and increased to 69.5. There was no discrepancy between sexes for the QoL. Age was the only sociodemographic criterion that affected the quality of life; older adults increased their QoL to 74.6. Young people went from 66.7 to 67.85. Spouses said that the app had a positive impact on their QoL. Regarding HL^h^, the average rate of 26.10 (in 40) increased to 30.68. Internet interventions can help caregivers meet their needs, which is a positive experience. Allowed to get to know their peers and to feel less lonely.	Caregivers can benefit from the VCoP because it enables interaction and knowledge sharing between caregivers and helps them meet their needs. VCoP’s impact is governed by age and relationship with the person cared for. It was positive for the caregivers’ quality of life, at a physical level, when the functional condition of the person with Alzheimer disease worsened. The VCoP was considered a useful tool. HL had a positive impact on the physical area of the QoL of caregivers.
First contact made by phone (data collection: demographics, health, “Deficit of quality-of-life technology” questionnaire, and CREATE^i^). Classified participants according to their HL level and portal use. This classification resulted in 4 groups (group 1: high HL, yes portal; group 2: high HL, no portal; group 3: low HL, yes portal; and group 4: low HL, no portal). Second contact made with 4 focus groups (N=75) aimed at analyzing participants’ attitudes. Sessions took 1 hour, were recorded, and used NVS^j^.	Participants with the higher HL who use the portal struggle to solve issues without the digital support and feel more pressured to use these methods. Those who do not use the portal say they do not feel safe using it due to the risk of sharing personal data and prefer to use the phone. People with low HL who do not use the portal do not have experience using computers, are not trained, and do not have internet access at their homes, but those who use the portal say they are more interested in learning and training with new technologies. People who are more familiar with accessing health information using the internet might be more willing to participate in research related to digital technology. The study revealed that HL was a factor that contributed to trust when accessing digital health information. However, it was not directly related to the motivation to get involved in health care. If portal users understand the benefits, this would be a motivation for portal use. Specific technology training is required to gain trust. ICs play a potential role in improving access to portal use for older adults who cannot access portals.	The study concluded that there should be more research focused on the attitudes and experiences of ICs of older adults as substitute users for the older adults. Health organizations should connect people to technology by adopting the following strategies: campaign to disclose the benefits of technology and how they meet people’s needs; offer specific training so that they can use technological tools in a secure, trustful way; include ICs in the campaign and training; and create workflows where people can communicate to update data, exchange information, and clarify any doubts that validate their knowledge. This would create a tool designed for support and commitment.

^a^ICT: Information and Communication Technology.

^b^IC: informal caregiver.

^c^SCD: sickle cell disease.

^d^AFMADO: Osona’s Association of Alzheimer’s Family Caregivers.

^e^CoP: community of practice.

^f^VCoP: virtual community of practice.

^g^QoL: quality of life.

^h^HL: health literacy.

^i^CREATE: Center for Research and Education on Accessible Technology and Experiences.

^j^NVS: Newest Vital Sign.

### Main Findings

Table 2 shows the main findings and conclusions of the studies described in the papers.

Regarding the year of publication, the studies were published in year 2017 (1/9, 11%); year 2019 (1/9, 11%); year 2020 (4/9, 44%); and year 2021 (3/9, 33%). They were conducted in the following countries: Canada (4/9, 44%), the United States (2/9, 22%), Scotland (1/9, 11%), Singapore (1/9, 11%), and Spain (1/9, 11%). Of the 9 studies, 3 (33%) followed a qualitative approach, 4 (44%) followed a mixed approach, 1 (11%) was observational, and 1 (11%) was a quasi-experiment.

The results of the studies enabled us to address the guiding questions. On the question “Does the Informal Caregiver have access to digital technology?” the studies show that ICs have access to and use digital technology [23,25]. They describe which types of technological resources are used more frequently by them: smartphones with mobile apps or internet access. The internet is the source of choice for accessing health information and learning about self-management techniques, with the importance of reliable websites being emphasized [22,25,26]. Smartphones are used to make calls, send SMS text messages and emails, and access the internet [25]. Apps are used to manage the appointments and medical information of the person cared for and to access specific information that allows ICs to share and discuss to commit to the caregiving relationship [25]. The esthetic dimension, ease of configuration, and nice interface are app features that are valued by ICs [25,26].

Privacy and security issues seem to be a factor that limits the use of technologies because users feel insecure due to the risk of sharing personal information [19,27]. Struggling to identify relevant and reliable information is also a factor that causes apprehension when it comes to internet use [26]. The degree of trust when accessing digital information seems to be related to the HL level of users [29]. Users with low HL levels who did not use the technology that was being analyzed had little experience using computers, no training, and no internet access at home. Those who used the portal showed increased interest in learning and practicing with the new technologies [29]. This fact reinforces the need for specific training on the use of digital technologies to gain trust [29].

Regarding the question “Does the Informal Caregiver use digital technology to improve their HL and their training in caring for the person cared for?” the studies show that the use of digital technology can benefit the population as well as caregivers [21,25-27,29].

ICTs lower information access barriers and provide relevant information that is enabled when there is a need to gain new knowledge [21,26]. ICTs are perceived as giving ICs the opportunity to guide themselves and interact with other caregivers, which allows them to get to know their peers and feel less lonely [24,28].

Caregivers say that by using digital technologies, they can obtain basic information about the disease, such as symptoms and treatment options, and about existing resources and equipment, which makes them feel less lonely in this role [21-23]. With the support of digital tools, caregivers felt recognized and heard and could express their uncertainties, stories, and suggestions, which increased their commitment to care provision [22]. Technology also maintains their caring ability and allows them to develop coping strategies [24].

The use of technology is also referred to as an intermediary for an improved quality of life [21,28]. This perception of the improvement of the quality of life is boosted when the technology that is used includes multiple resources, such as the fact that there is a public forum for community discussion with other ICs in the same region without having to leave home, a market to purchase and sell materials and equipment that is needed for providing care, and an alert system or information trackers [26,27]. The integration of the multiple resources that are needed by caregivers daily in an app that is easy to use allows them to save time and provide help to browse without problems [27].

Another aspect referred to by the studies concerns suggestions or factors that can improve the experience of ICs when using digital technologies. One study described that new technologies that are only focused on the task can lose their value as they lose the capacity to provide information that is relevant to caregivers’ needs [21]. It is important that ICs collaborate in the development of technologies because their deep knowledge of the needs and existing gaps contribute to technological innovation, which allows them to overcome the existing technological barriers and learn facilitator mechanisms [23].

The expectations of ICs as to digital technologies also seemed to be an important aspect to consider because they can increase the technology used. ICs hope that technologies can provide emotional and psychological support, informative support from the family, training and education, and health care follow-up [23,26]. In a more practical way, ICs suggested that there should be resources that help them with their mental health, namely, relaxation techniques and motivational quotes and guides that would help them remember to take care of themselves [27].

## Discussion

### Informal Caregivers’ Role in the Health Care System

According to the studies that were analyzed, demographic changes are leading to an increasing need for long-term care, which results in people informally caring for their relatives. Being an informal or family caregiver brings uncertainties, isolation, and overload [21,27]. Studies have shown that the involvement of the caregiver in the care plan is essential. The active involvement of ICs as a member of the interprofessional care team results in an improved experience, increased caregiver capacity, and the appreciation of the caregiver role [22,24].

These results are in accordance with the literature where ICs are considered “one of the elements of the sustainability of social and health systems” [30]. This emphasizes how important it is for health care professionals to work with ICs to find the strategies that are most adequate for effective empowerment [30]. The empowerment of ICs should be “a priority in health care organizations and the nurse assumes a major, dynamic, empowering role when it comes to the most adequate response to meet those needs” [31].

The World Health Organization (WHO) has defined a long-term strategy for the expansion and use of digital health, emphasizing the positive impact that it can have on health care access and provision as well as on the health and well-being of the population and caregivers [23]. According to the literature, health technology is “one of the strategies used by the health care professional to empower citizens to use it in a secure way” [32].

### The Use of Digital Technologies Supporting Caregivers

The studies revealed that low HL was a barrier to accessing digital information and the correct use of technological tools. Lack of training makes browsing difficult and results in user insecurity [26,29]. The initial findings of a European survey on population HL carried out by the WHO Action Network on Measuring Population and Organizational Health Literacy indicate that 22% to 58% of the population find it challenging to access and interpret digital health information [33]. By contrast, the European data report shows that, in 2019, in European countries such as Finland, the Netherlands, the United Kingdom, and Germany, 75% of the active population had basic digital skills [34].

Promoting HL improves safety in caregiving and decreases the risk associated with this activity [32].

Using digital technology in the health field can benefit the caregivers and the general population [21,25-27,29].

Questions about privacy and security when using these digital tools are an important factor for users. Although there is an increasing concern about what is the best way to develop emerging web-based technologies (eg, ethical data use), the results show that a hybrid model with a web-based and in-person approach can work well for caregivers in rural areas [23]. The model that includes digital technology and an in-person approach is pointed out as a more reliable model for the ICs.

These results are in line with the American study that described that ICs use the internet (77.5%) to access health information for themselves (73%), for others (67.5%), and to communicate with the physician [35].

The results highlight that ICs intend to use digital tools to establish communication relationships with people cared for, their family members, the peers, and health care professionals [21,23,24,27,28].

### Principal Findings

The text highlights privacy concerns limiting technology use, underscoring the impact of low HL on users’ digital engagement. ICs benefit from digital tools by experiencing empowerment, recognition, and an improved quality of life. The integration of multiple resources in one technological tool supports caregiving, saving time and facilitating daily tasks. The collaboration of ICs in technology development is crucial for innovation and overcoming barriers, emphasizing the need for user-driven solutions.

### Limitations

As for the analysis of the included studies, it was not possible to use a tool to evaluate study quality. In the papers that were analyzed, it was not possible to identify references about improvement opportunities arising from the research process. The fact that the samples in the presented studies are small does not allow us to extrapolate data to the population.

The included papers were published in English, French, Spanish, and Portuguese, and the inclusion of articles in other languages could have brought more relevant information to this review. However, searching in 4 databases allowed us to expand the search comprehensiveness.

### Comparison With Previous Work

In Portugal, there are few scientific studies carried out by nurses that refer to the use of digital technologies as a resource to train people with dependence and ICs.

### Conclusions

Evidence found in studies revealed that ICTs such as digital platforms, portals, and web-based community groups were preferentially used by informal caregivers via mobile apps and that computers were used more by the people cared for. Studies showed that ICs had access to and used digital technology not only to meet the needs of the person cared for but also to meet their own needs. Studies have shown that digital technology is an accessible tool for empowering ICs. However, there were concerns regarding privacy, security, and the use of these tools, which should be considered by health care professionals and researchers. It is also important to highlight the necessity of providing digital training for both ICs and the individuals under their care.

ICs play a key role in the provision of quality care to the dependent people to whom they commit. It is crucial to understand how digital tools can be effectively and beneficially used to empower ICs.

The participation of ICs is essential when it comes to developing digital tools (platforms, mobile apps, and portals) because they can contribute to developing tools that meet users’ needs (ICs and the people cared for). The use of digital technologies can guarantee access to knowledge, thereby empowering caregivers when it comes to making a decision and sharing care provision with health care professionals. It is important to emphasize the significance of digital empowerment in enhancing the digital health literacy of both ICs and those they care for. Digital technology allows accessible, targeted, and effective communication. Health care professionals and researchers should guarantee information reliability, security, and clarity and optimize existing resources.

## Appendix

Multimedia Appendix 1 PRISMA-ScR checklist.

## Abbreviations

HL: health literacy
IC: informal caregiver
ICT: Information and Communication Technology
JBI: Joanna Briggs Institute
PRISMA: Preferred Reporting Items for Systematic Reviews and Meta-Analysis
WHO: World Health Organization
